# Pelvic Inflammation

**Published:** 1887-04

**Authors:** W. E. Ewing


### PELVIC INFLAMMATION.

BY W. E. EWING, M. D.

[Read before the Nashville Academy of Medicine and Surgery.]

In order to thoroughly appreciate the pathology of pelvic inflammation it is necessary to keep prominently in view the anatomy of the pelvic organs. Within the pelvis there is an abundant supply of connective or cellular tissue. It is found to a greater or less extent in all the intervening spaces between the various organs contained in the pelvic cavity, except between the peritoneum and posterior, superior and anterior surfaces of the body of the uterus. It exists in greatest abundance at the junction of the broad ligaments with the uterus and around the cervix, and is found in considerable quantity between the folds of the broad and utero-sacral ligaments. In addition to acting as a bond of union between these structures, it is the channel through which the blood-vessels pass.

The peritoneum invests all the pelvic structures. It descends into the pelvis in front of the rectum, covers a small part of the posterior wall of the vagina, and is then reflected on to the uterus, the fundus and body of which it covers. From the anterior surface of the uterus it is reflected upon the posterior wall of the bladder. From the sides of the uterus the peritoneum reflected on each side to the wall of the pelvis, forming the broad ligaments, between the layers of which, in addition to the cellular tissue already mentioned, is found blood-vessels and lymphatics; the fallopian tubes are situated in the upper free margin of the broad ligaments (and are covered by peritoneum except at the fimbriated extremity). The fallopian canal begins at the uterus and terminates at the peritoneal cavity, thus creating a closer communication between the vagina, uterus, fallopian tubes and peritoneum; the ovaries are situated on the sides of the uterus in the posterior wing of the broad ligaments, just behind the fallopian tubes.

The literature upon the subject of pelvic inflammations has been very extensive, but unfortunately the theories advanced as to the pathological site have been at such variance that we have not gained as much information upon the subject as is generally gained upon affections receiving so much attention.

Nonat, in his work, while acknowledging such an affection as pelvic peritonitis, locates the site of the inflammation chiefly in the cellular tissue. Beneutz and Gaupil occupy a position directly opposed to the one assumed by him; whilst not denying the existence of cellulitis in their treatise upon diseases of women, locate the affection chiefly in the peritoneum, regarding it almost as a condition synonymous to orchoitis in the male.

While I am ready to admit that it is possible to have a slight inflammation confining itself to either cellular tissue or peritoneum, I cannot conceive how it is possible—looking at the anatomical relations of these structures—for an extensive cellulitis to exist without the peritoneum becoming sooner or later involved to some extent, and *vice versa.* The predominating inflammation is sometimes, doubtless, situated in the cellular tissue and sometimes in the peritoneum. The latter, in my opinion, is much oftener met with, and is more chronic in nature, producing viscereal adhesions, the result of false membranes, and that the tumors are frequently formed by matting together of the various intra-pelvic viscera as a consequence of the inflammation; whereas, when the predominating inflammation is in the cellular tissue, it is more likely to run a regular course.

### ETIOLOGY.

Pelvic inflammation is secondary, whether located in one structure or another, being the result of septic absorption, or the extension of inflammation from the fallopian tubes or ovaries.

A greater number of cases are credited to parturition and absorption than to any other source, and it is the result of septic absorption of the lymphatics.

Next in the order of frequency, and in importance, is gonorrhœa, which produces it by an extension of the gonorrhœal virus from a vaginitis to the uterine cavity, from thence to and through the fallopian tubes to the peritoneum, thus setting up peritonitis. In puerperal women gonorrhœa is of special interest as to the causation of this affection.

According to Noggeerath, a common cause of pelvic inflammation is what he terms latent gonorrhœa in the male, he being of the opinion that a gonorrhœa once contracted is never entirely cured, and that it is capable of communicating its specific infection years after. Noggeerath's views are doubtless extreme, for the number of cases of previous gonorrhœa in the male is out of proportion to the number of women who suffer with pelvic inflammation ; but his views are of service, for they assist in impressing the fact that to gonorrhœa is due many cases of pelvic inflammation. Imprudence during menetruation produces many cases, and when we take into consideration the minuteness of the fallopian canal, especially near its uterine extremity, we can easily appreciate how inflammation would result secondarily from the engorgement which sometimes accompanies sudden suppression of the menstrual flow. The number of other causes, such as ovaritis, excessive venery, traumatic issues, etc., are so numerous that we will not trespass upon your time to speak of them, thinking we have already dwelt long enough upon the etiology.

### PATHOLOGY.

The pathology differs with the structure chiefly involved, as well as with the location and stage of the disease. If the inflammation is predominating in the cellular tissue the same changes will be met with that are in existence in an ordinary phlegmon, viz : first, congestion ; second, effusion ; third, suppuration ; frequently, however, it does not pass through the three stages, as it may be arrested in the first, or end in resolution in the second stage. Again, it may neither end in resolution nor suppuration, but by absorption of the serous portion of the liquor sanguinus, and the organization of the plastic lymph may result in permanent induration of the affected parts. When the peritoneum is chiefly involved, if acute, there is little or no difference in the pathology from the one just given. If the disease is subacute or chronic, the exudation, which is plastic, produces the matting together of the pelvic-viscera, the fallopian tubes and ovaries, as well as the uterus, being frequently displaced and immovable.

### COMPLICATIONS.

The most important complications are ovaritis and salpingitis.

### SYMPTOMS.

In acute attacks pain is usually one of the first symptoms, and this is generally attended by a chill, following which there is an increase in temperature, a full, bounding pulse ; in severe cases there is more or less tympanitis, great tenderness in hypogastric and iliac regions ; the patient will lie upon her back with the thighs flexed, and the bladder and rectum are generally affected by reflex action, the bladder becoming so irritable that there is a frequent desire to micturate; constipation is the rule. When the disease is chronic the symptoms are not so prominent ; the pain is less acute, neither is it attended with the inflammatory symptoms that are sometimes so obscure that the disease is discoverable only by physical examination.

### PHYSICAL SIGNS.

A vaginal examination during the first stage of an attack will find a marked local elevation of temperature, and the parts excessively tender; a little later, after exudation has taken place, a fullness is discovered, and, as the disease progresses, the uterus becomes fixed in its position, frequently displaced; a tumor is often discoverable in some portion of the pelvic cavity, usually to the side or posterior to the uterus. These tumors may be abscesses, displaced ovaries, or merely an agglutination of the pelvic viscera.

As the chronic is frequently the sequel of an acute attack, an examination at this stage would reveal the same condition of things that is found to exist in the latter part of the acute attack, which has just been given.

### DIAGNOSIS.

The only affection that is likely to be mistaken for pelvic inflammation is pelvic hæmatocele, from which it can be distinguished by the symptoms of inflammation in the one, and symptoms incident to hemorrhage in the other. The tumor met with in hæmatocele is, as a rule, posterior to the uterus, while that resulting from pelvic inflammation oftimes exists upon the side of the uterus. The location of the tumor, however, can not be relied upon. The tumor ensuing from the inflammation is hard at first and either disappears or softens as suppuration ensues.

### TREATMENT.

If the patient is seen early, ahe must be kept perfectly quiet in bed; pain must be relieved by morphia, administered hypodermically; afterwards the pain should be kept subdued by suppositories of morphia, administered as often as may be necessary; warm poultices should be applied over the abdomen; hot water vaginal injections should be administered freely. It is claimed by Emmet that hot water injections, resorted to at the beginning of an attack, will frequently cut short the inflammation. The bowels, in the early stage of the disease, should not be interfered with: as the disease progresses, however, they should be kept regular with mild aperients. The febrile excitement, if high, should be controlled by aconite. The treatment in the second stage varies little from that resorted to during the congestive period, excepting that a large plaster over the lower part of the abdomen will now be of service. If suppuration ensues—which is indicated by signs—followed by fever and increase of pain, tonics, such as iron and quinine, will be demanded, along with the most nourishing diet. The diet should be looked after from the beginning; in the early stage it should be chiefly milk; as the strength diminishes, in addition to the milk, beef tea, soups, etc., should be added, and stimulants may be required to sustain the patient. Should an abscess form, it should be immediately evacuated, and the place of opening should be the point indicated by Nature, it matters not whether that be through the vagina, rectum or abdominal wall.

When adhesions are present, unless very extensive, it is better to leave the case to Nature. instructing the individual as to the precautions necessary to prevent relapses. Should, however, the ovaries or fallopian tubes be diseased to such an extent as to threaten the life of the patient, or if by constant recurrence of the inflammation the woman's suffering is such as to make life a burden, laparotomy should be resorted to.

